# Infodemics and Vaccine Confidence: Protocol for Social Listening and Insight Generation to Inform Action

**DOI:** 10.2196/51909

**Published:** 2024-10-24

**Authors:** Jessica Kolis, Kathryn Brookmeyer, Yulia Chuvileva, Christopher Voegeli, Sarina Juma, Atsuyoshi Ishizumi, Katy Renfro, Elisabeth Wilhelm, Hannah Tice, Hannah Fogarty, Irma Kocer, Jordan Helms, Anisha Verma

**Affiliations:** 1 Global Immunization Division Global Health Center Centers for Disease Control and Prevention Atlanta, GA United States; 2 Office of the Director National Center for HIV, Hepatitis, STD, and TB Prevention Centers for Disease Control and Prevention Atlanta, GA United States; 3 Division of Population Health National Center for Chronic Disease Prevention and Health Promotion Centers for Disease Control and Prevention Atlanta, GA United States; 4 Office of the Director National Center for Immunization and Respiratory Diseases Centers for Disease Control and Prevention Atlanta, GA United States; 5 Division of Workforce Development National Center for State, Tribal, Local, and Territorial Public Health Infrastructure and Workforce Centers for Disease Control and Prevention Atlanta, GA United States; 6 Division of STD Prevention National Center for HIV, Hepatitis, STD, and TB Prevention Centers for Disease Control and Prevention Atlanta, GA United States; 7 Division of State and Local Readiness Office of Readiness and Response Centers for Disease Control and Prevention Atlanta, GA United States; 8 Division of Overdose Prevention National Center for Injury Prevention and Control Centers for Disease Control and Prevention Atlanta, GA United States; 9 Division of HIV Prevention National Center for HIV, Hepatitis, STD, and TB Prevention Centers for Disease Control and Prevention Atlanta, GA United States; 10 Tanaq Support Services Atlanta, GA United States

**Keywords:** infodemic, infodemic management, vaccine confidence, vaccine demand, misinformation, disinformation, infodemiology, mixed methods, thematic analysis, COVID-19

## Abstract

**Background:**

In the fall of 2020, the COVID-19 infodemic began to affect public confidence in and demand for COVID-19 vaccines in the United States. While polls indicated *what* consumers felt regarding COVID-19 vaccines, they did not provide an understanding of *why* they felt that way or the social and informational influences that factored into vaccine confidence and uptake. It was essential for us to better understand how information ecosystems were affecting the confidence in and demand for COVID-19 vaccines in the United States.

**Objective:**

The US Centers for Disease Control and Prevention (CDC) established an Insights Unit within the COVID-19 Response’s Vaccine Task Force in January 2021 to assist the agency in acting more swiftly to address the questions, concerns, perceptions, and misinformation that appeared to be affecting uptake of COVID-19 vaccines. We established a novel methodology to rapidly detect and report on trends in vaccine confidence and demand to guide communication efforts and improve programmatic quality in near real time.

**Methods:**

We identified and assessed data sources for inclusion through an informal landscape analysis using a snowball method. Selected data sources provided an expansive look at the information ecosystem of the United States regarding COVID-19 vaccines. The CDC’s Vaccinate with Confidence framework and the World Health Organization’s behavioral and social drivers for vaccine decision-making framework were selected as guiding principles for interpreting generated insights and their impact. We used qualitative thematic analysis methods and a consensus-building approach to identify prevailing and emerging themes, assess their potential threat to vaccine confidence, and propose actions to increase confidence and demand.

**Results:**

As of August 2022, we have produced and distributed 34 reports to >950 recipients within the CDC and externally. State and local health departments, nonprofit organizations, professional associations, and congressional committees have referenced and used the reports for learning about COVID-19 vaccine confidence and demand, developing communication strategies, and demonstrating how the CDC monitored and responded to misinformation. A survey of the reports’ end users found that nearly 75% (40/53) of respondents found them “very” or “extremely” relevant and 52% (32/61) used the reports to inform communication strategies. In addition, our methodology underwent continuous process improvement to increase the rigor of the research process, the validity of the findings, and the usability of the reports.

**Conclusions:**

This methodology can serve as a diagnostic technique for rapidly identifying opportunities for public health interventions and prevention. As the methodology itself is adaptable, it could be leveraged and scaled for use in a variety of public health settings. Furthermore, it could be considered beyond acute public health crises to support adherence to guidance and recommendations and could be considered within routine monitoring and surveillance systems.

## Introduction

### Background

Responding to the unprecedented COVID-19 pandemic has been made even more challenging by an extraordinary infodemic, defined as an overabundance of information, including credible and false or misleading information, during a disease outbreak [1]. Information voids (ie, lack of accurate information on a specific topic from credible sources), misinformation (ie, inaccurate information), and disinformation (ie, inaccurate information designed to achieve an agenda) have been hallmarks of the COVID-19 infodemic [2-4]. Furthermore, information voids left by evolving science and rapidly spreading misinformation have served as breeding grounds for public confusion [3], posing a serious risk to compliance with public health prevention and the uptake of mitigation measures, such as mask wearing and vaccination efforts [4,5].

In the fall of 2020, the influence of the infodemic on public confidence in COVID-19 vaccines was evident before any vaccine was granted an emergency use authorization. National polls and surveys conducted in September 2020 and December 2020 indicated wide-ranging levels of public confidence in COVID-19 vaccines and their rollout [6]. In December 2020, before any COVID-19 vaccine was authorized for emergency use, more than a quarter of those surveyed said that they would not get vaccinated, citing concerns about the side effects and safety, lack of trust in the government, and worries about the speed at which vaccines were developed [7]. In addition, in December 2020 and January 2021, two-thirds of adults in the United States (65%) said that the federal government was doing a “fair” or “poor” job of distributing vaccines [7,8]. While these large-scale surveys signaled *what* Americans felt about COVID-19 vaccines, they did not provide an understanding of the reasons *why* they felt that way and the social and informational influences that factored into vaccine confidence and uptake.

### Creating the Centers for Disease Control and Prevention Insights Unit

In late 2020, the leadership of the US Centers for Disease Control and Prevention (CDC) COVID-19 Response recognized that the agency needed to act more swiftly to address the questions, concerns, perceptions, information voids, and circulating mis- and disinformation that affected people’s health decision-making, particularly regarding COVID-19 vaccines. At that time, there was no established, coordinated mechanism for collecting, reviewing, and synthesizing qualitative and quantitative data from multiple CDC-owned and external sources to assess the state of vaccine confidence in the United States.

The Insights Unit was established in January 2021 and became the first infodemic management unit of its kind at the CDC. The Insights Unit was part of the Vaccine Confidence and Demand Team within the Vaccine Task Force (VTF) of the CDC’s COVID-19 Response. The Insights Unit established a novel approach and methodology to rapidly detect trends in vaccine confidence by integrating multiple data sources and using established frameworks to understand the knowledge, attitudes, and behaviors of Americans regarding the COVID-19 vaccines. The aim was to use this methodology to help inform communication and programmatic strategies at a national level through a continual and iterative listening and feedback process [9].

The initial Insights Unit was a 3-person, multidisciplinary team led by a health communication specialist with experience in immunization campaigns and a behavioral scientist, with additional support from a data analyst with a public health background. The unit remained multidisciplinary and ranged in capacity between 1 to 2 full-time unit leads and 1 to 6 full- and part-time data analysts during the first year.

### Manuscript Aims and Goals

This manuscript aims to outline the Insights Unit’s first year and the establishment, approach, and methodology for COVID-19 State of Vaccine Confidence (SoVC) Insights Reports so that others can replicate and adapt its processes and methods. We hope to help health authorities and public health professionals explore opportunities for establishing insights units or complex social listening mechanisms for any public health area of concern. They can use these units and their findings to examine information ecosystems to gain insights into how their community’s thoughts and feelings affect critical health decisions and use it to enact policies, design and adapt programs, and inform communication campaigns.

## Methods

### Analysis Plan Development

#### Planning

##### Landscape Analysis

Before the establishment of the Insights Unit, there were several social listening activities about COVID-19 being conducted by the US government and external organizations. Many of the activities, even within the CDC, were being conducted and interpreted independently with little to no synthesis or coordination. We conducted an informal landscape analysis in January 2021 to identify and evaluate potential data sources (primary and secondary) for inclusion in our reports. We identified data sources by interviewing colleagues via a snowball sampling strategy within the COVID-19 Response and across the CDC with colleagues recommending others for us to interview about potential sources for inclusion. Some interviews were conducted via phone, whereas others were conducted over email. The interviews remained informal with no standard set of questions for each colleague interviewed; however, each had the same objective of identifying what social listening data or sources they used, conducted or created, or were aware of, internal or external to the CDC. These informal interviews and emails helped us identify all currently available social listening data sources within the agency and better understand what types of data sources they used in social listening and monitoring efforts.

The identified data sources were then categorized as mixed methods reports (ie, reports that included multiple primary data sets or multiple types of data sets, such as social media and news media monitoring), social listening (ie, data collected from social media aggregation tools or native platform searches), direct reports (ie, primary data sets, such as a media request line list or a CDC-INFO inquiry line list), and research (eg, recently published peer-reviewed and gray literature and polls and surveys from accredited institutions and organizations). We informally assessed 15 identified data sources for accessibility, suitability, and methodological rigor (see Textbox 1, which includes both primary [ie, data collected directly by the CDC that could be analyzed by our analysts] and secondary [ie, reports of data already collected and analyzed by analysts outside the Insights Unit] data sources). The first evaluation criteria focused on accessibility, where we considered where or how we could access the data, whether primary or secondary; how frequently data sets were made available; and whether there was a cost or subscription needed to access data (eg, Meltwater [Meltwater News US Inc]). After accessibility was assessed for each source, we considered the data source’s suitability and whether it would be able to help us understand vaccine confidence. Finally, we considered the methodological rigor of each data source, including considering sampling strategies for primary data and analytic approaches for secondary data sources. The first report, produced less than a month after the creation of our unit, included data from 11 sources from the initial landscape analysis. Our application of evaluation criteria became more rigorous as the unit expanded and we better understood our data use and the quality of data sources.

After the initial inclusion criteria were evaluated, we classified each source into one of the following categories: mixed methods reports, social media listening, direct reports, and research. We then considered the type and amount of data within each source to determine appropriate techniques for data collection and analysis and clarified what the data would be able to tell us in relation to vaccine confidence (Table 1).

**Evaluation criteria for data sources—criteria categories and their definitions that were used to evaluate each potential data source before inclusion in social listening and analysis.**

**Accessibility**

- How easily the unit could access the data or the findings
- How frequently the data or results were made available
- The cost, if applicable, associated with accessing the data

**Suitability**

- Comparing the available data with the intended purpose of reporting on the state of vaccine confidence

**Methodological rigor**

- Data collection methods used in pulling the data
- Analytic approaches used when reports presented findings

**Table 1:** Data source categories and intended use^a^.

Data source type	Definition	Use
Mixed methods reports	Reviewed multiple primary data sets Included multiple types of data sets (ie, social media listening and news media monitoring)	Trending and emerging topics and keywords Changes in information-seeking patterns Understanding sentiment Information gaps and voids Sociobehavioral indicators Vaccination barriers Misinformation narratives
Social media listening	Data collected from social media aggregation tools or native platform searches	Conversation levels by topics (ie, number of posts and level of engagement with posts) Share of voice by topic or subtopic and percentage of total conversation occupied by a single topic or subtopic Trending and emerging topics and keywords Changes in information-seeking patterns Understanding sentiment Information gaps and voids Sociobehavioral indicators Vaccination barriers Misinformation narratives
Direct reports	Primary data sets (eg, media request line list or CDC^b^-INFO inquiry line list)	Conversation levels by topics (ie, number of posts and level of engagement with posts) Share of voice by topic and subtopic Trending and emerging topics and keywords Changes in information-seeking patterns Information gaps and voids Vaccination barriers Misinformation narratives
Research	Recently published peer-reviewed and gray literature Internal and external polls from accredited institutions and organizations	Identify sociobehavioral indicators Determine vaccination intent Identify vaccination barriers

^a^The data sources used were classified into 1 of the 4 categories (mixed methods reports, social media listening, direct reports, and research), and their potential use approaches were identified based on those categories. These approaches outlined what type of analyses might be used and what issues they might be able to elucidate.

^b^CDC: Centers for Disease Control and Prevention.

##### Analysis and Reporting Cadence

**Figure 1:**
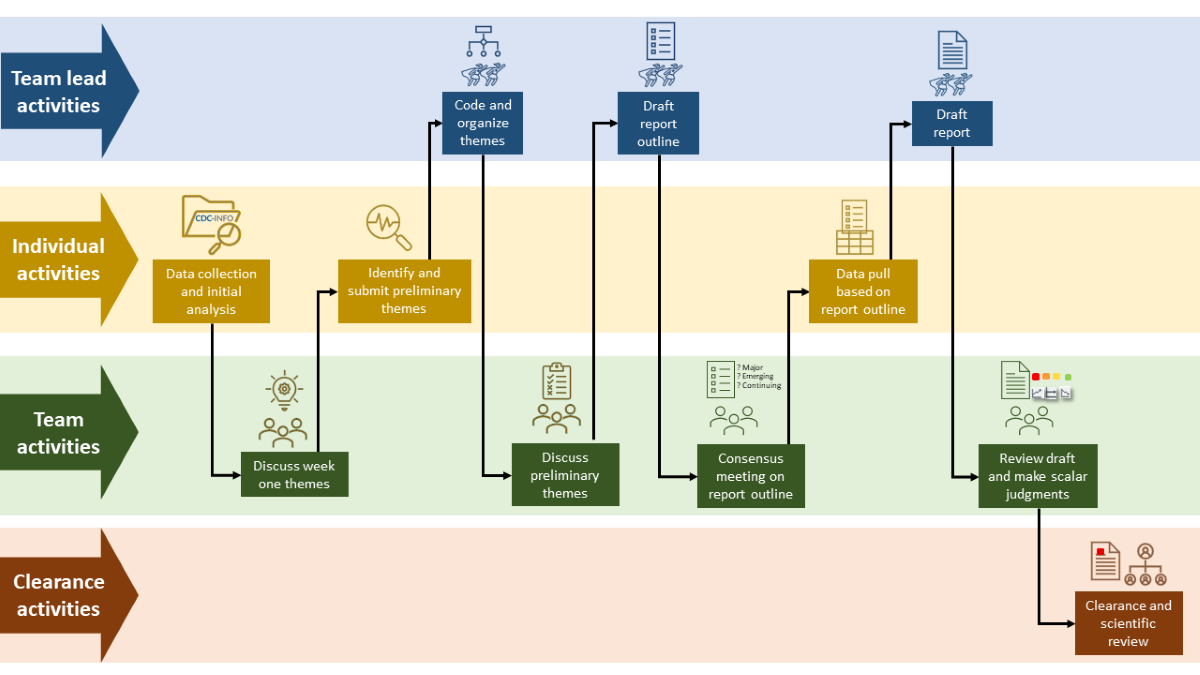
Visualization of report production. Reports went through a multistep process with phases that occurred at separate levels (eg, team lead, individual analyst, team, and clearance).

During the Insights Unit’s first year (January 2021-January 2022), the team created and disseminated SoVC reports biweekly. Data collection and analysis occurred concurrently with report production and clearance, which ensured that the reports provided insights on emerging themes quickly and without gaps in data collection. Analysts performed data collection and analysis for their assigned data segments for 2 weeks and submitted preliminary findings to unit lead(s). Unit lead(s) then led multiple consensus-building meetings to develop the report outline, draft the report narrative, and complete the scientific review (Figure 1 and Multimedia Appendix 1).

#### Social Listening and Integrated Analysis

##### Analytical Frameworks

**Figure 2:**
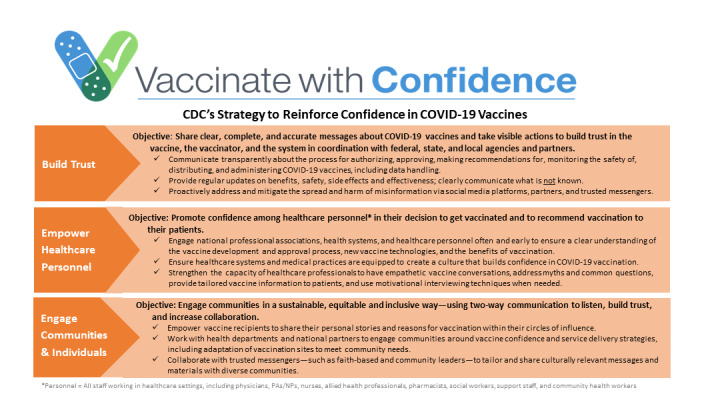
Centers for Disease Control and Prevention (CDC) Vaccinate with Confidence strategy for COVID-19 vaccines. Vaccinate with Confidence is the strategic framework of the CDC to strengthen confidence in COVID-19 vaccines through 3 strategies: building trust, empowering health care personnel, and engaging communities and individuals. NP: nurse practitioner; PA: physician assistant. Soure: CDC [10].

We considered 2 key frameworks in the development of our approach for analyzing and interpreting our collected data. The first was the CDC’s Vaccinate with Confidence strategy, which was created to bolster confidence in COVID-19 vaccines (Figure 2) [10]. The second was the World Health Organization’s behavioral and social drivers for vaccine decision-making framework, which includes 4 domains that influence vaccine demand and uptake [11].

**Figure 3:**
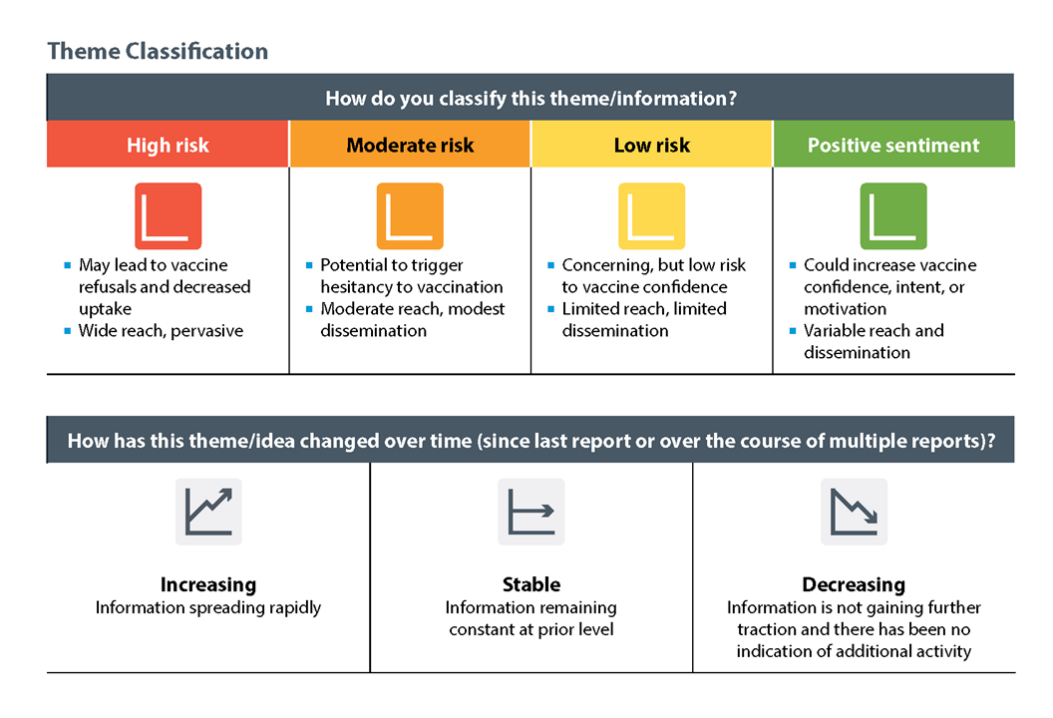
COVID-19 State of Vaccine Confidence Insights Report threat matrix. The Centers for Disease Control and Prevention COVID-19 Insights Unit developed a threat matrix to help denote the potential threat that an identified theme would have on one’s intent to vaccinate. Themes were classified by level of threat to vaccination (eg, high risk of impacting vaccination) and directionality, which indicated the relative volume and prevalence of a theme across information systems.

On the basis of these frameworks and informed by the United Nations Children’s Fund Vaccine Misinformation Management Field Guide [12], as well as the UK Government Communication Service’s Recognize Mis- and Disinformation, Early Warning, Situational Insight, Impact Analysis, Strategic Communication, Tracking Effectiveness counter-disinformation toolkit [13], a threat matrix was developed to provide further context to our findings and determine possible actions to address dominant and emerging vaccine confidence issues (Figure 3).

##### Data Segment Analysis

Analysts were assigned to at least one designated data segment. Data segments were grouped as categories of data and included external social listening (ie, social media broadly), internal social listening (eg, engagement on CDC-owned social media platforms and calls or email inquiries to CDC-INFO), media monitoring (ie, news media monitoring and media request line list), research (ie, literature and surveys), and internet trends (eg, Google Trends and website traffic). Each analyst was responsible for data collection and preliminary analysis of their assigned data sources. Analysis methods varied slightly depending on the data source category and how the data source could be used based on the previous evaluation (Table 2). All analyses used established qualitative theme identification techniques [14] (Textbox 2). For data segment analysis, the techniques of repetition, such as looking for high volume of key terms of phrases in inquiries to CDC-INFO or mentions in news media, and indigenous categories, such as looking for unique terms or phrases such as “viral shedding,” were predominantly used to identify preliminary themes (Table 2) [15]. In addition, analysts used a mixed deductive and inductive approach to identify preliminary themes. Deductively, analysts organized the data into major categories represented by the aforementioned analytical frameworks: vaccines, vaccinators, vaccine system, and outside the vaccine system (Table S1 in Multimedia Appendix 2). Inductively, they allowed themes to emerge organically, not allowing the assembled codebook to limit their findings and potentially missing new emerging themes, topics, or terms. Following initial identification, analysts further examined their identified themes to determine whether an individual theme was a single development with a loud “echo” (eg, multiple media sources around the country reported on the same new workplace vaccination policy rolled out by a company and repeated posts, inquiries, and searches about that particular policy) or a repeated development with multiple iterations of the theme across the data segment (eg, different media sources around the country reporting on multiple companies rolling out similar workplace vaccination policies and conversations online amplified each of the individual policies separately) and documented which type it was.

**Table 2:** Data segment collection and analysis^a^.

Data segment and source					Collection method			Additional criteria			Analysis method
**Primary data**											
	**News media^b^**										
		Meltwater^c^	Boolean search string (Table S2 in Multimedia Appendix 3)			English only United States only			Identify top and unique keywords in headlines and copy through established Boolean search strings by number of mentions Identify themes by looking for repetition following sorting mentions by reach as calculated by Meltwater until saturation (extremely high volumes meant that review of all lines of data were impossible given the time frame) Focused searches using top and unique keywords via snowball methods for context determination		
		CDC^d^ media office	Weekly line list			Inquiries routed to COVID-19 Vaccine Task Force			Identify themes based on top and unique keywords Determine directionality of emerging themes based on share of voice week to week		
	**External social listening^e^**										
		Meltwater^c^ CrowdTangle^f^	Boolean search string			English only			Identify top and unique keywords in headlines and copy through established Boolean search strings Identify themes by looking for repetition following sorting mentions by reach as calculated by Meltwater until saturation (extremely high volumes meant that review of all lines of data were impossible given the time frame) Focused searches using top and unique keywords via snowball methods for context determination		
	**Internal social listening^g,h^**										
		Facebook Twitter (subsequently rebranded X) Instagram	Native platform searches			—^i^			Identify top keywords and unique keywords within comments or post interactions on CDC-owned social media channels		
		CDC-INFO^j^	Weekly line list			Contains “COVID-19” and “vaccine” or “vaccinate” Duplicates removed			Random sample of 10% of the questions to identify themes in the questions Identify top and unique keywords within samples		
	**Research and academics**										
		Peer-reviewed and preprint literature	PubMed LitCovid Google Scholar			Published or made available during the reporting period Focus on the US population at national, state, or other jurisdictional levels Opinion pieces excluded			Articles reviewed to determine whether findings were related to variables within the BeSD^k^ framework and the CDC’s Vaccinate with Confidence strategy for COVID-19 vaccines		
		Polls and surveys	National Immunization Survey Kaiser Permanente Foundation Google			Data collected align with indicators in the BeSD framework and the CDC’s Vaccinate with Confidence strategy for COVID-19 vaccines Polls collected or published during the reporting period			Longitudinal polls reviewed to see changes in vaccine confidence over time and achieve point-in-time estimates for the reporting period Polls evaluated for focus on specific populations or geographic areas		
	**Web metrics^l^**										
		Internal web traffic	Adobe Analytics			—			Identify most frequently viewed CDC web pages and frequently asked questions Identify changes in weekly top search terms		
		External web searches	Google Trends			Data from Google Trends using an automated web scraping package available in the R software (R Foundation for Statistical Computing) looking at rising search queries and topics related to a prespecified set of keywords across Google and YouTube			Power BI^m^ (Microsoft Corp) was used to create tree map charts (visual representation of search interest data using chart size dimensions proportional to percentage increase) for quickly identifying keywords or topics experiencing the largest increase in search frequency on a given day		
**Confirmatory data segments**											
	Web metrics			Semrush			—			Semrush was used for targeted analysis of specific topics once themes were identified through primary sources	
	Third-party reports			COVID-19 Joint Information Center communication surveillance report Federal Emergency Management Agency Social Listening Report The Virality Project Project Vector NCIRD^n^ social listening report First Draft News Vaccine Misinformation Insights Report CrowdTangle content insights report			Reports using data from or published during the reporting period			Identify themes based on top and unique keywords and themes Used as a means to validate findings after triangulation to avoid double counting	

^a^All data sources were categorized into segments with similar data. The table shows what each data segment comprised and lists the exact source, how data were collected and analyzed, and whether there were additional inclusion and exclusion criteria.

^b^Due to the high volume of data, it was not possible to review every data point. Analysts reviewed data points until new themes could no longer be identified having reached a point of data saturation.

^c^Social listening aggregator tool.

^d^CDC: Centers for Disease Control and Prevention.

^e^Public posts on social media not interacting directly with CDC-owned social media channels on Twitter (subsequently rebranded X), Facebook, Instagram, Pinterest, Reddit, and YouTube.

^f^A public insights tool from Meta.

^g^Public posts on social media interacting directly with CDC-owned social media channels or direct inquiries to the agency via CDC-INFO.

^h^Due to the high volume of data, it was not possible to review every data point. Analysts reviewed data points until new themes could no longer be identified having reached a point of data saturation.

^i^Not applicable.

^j^The CDC’s national contact center. CDC-INFO offers live agents by phone and email to help people find the latest reliable and science-based health information on >750 health topics.

^k^BeSD: behavioral and social drivers for the uptake of vaccines.

^l^Analyses of web search data sought to understand trends in search activity, which can be indicators of emerging concerns, questions, or information voids.

^m^Data visualization software.

^n^NCIRD: CDC’s National Center for Immunization and Respiratory Diseases.

**Applied qualitative data analysis methods. Analysts used 4 key types of qualitative analysis techniques to identify preliminary themes. This textbox presents the different techniques used and how they were defined for our use.**

**Techniques and definitions**

- Repetition: themes that come up repeatedly in a single input or across our data inputs
- Similarities and differences: data points that differ or are like each other (which may make up subthemes to a larger overarching theme), themes that are like others that were presented in previous reports (continuing themes), or themes that are similar but with different elements from others that were presented in previous reports (evolving themes)
- Indigenous categories: technical or slang-sounding terms that are used in new or unique ways by the community under study, such as “viral shedding” or “medical segregation”
- Missing data: what should or could be talked about but is not, such as when the media report on a development but social media is quiet about it or a discussion about a development is missing critical information that exists (helping us identify information gaps)

##### Integrated Thematic Analysis

For each reporting period, analysts submitted preliminary findings from their assigned data segment or segments in a spreadsheet before the first consensus-building meeting. Each analyst could identify and submit an unrestricted number of preliminary themes. Each line of the spreadsheet contained one preliminary theme with a high-level summary of its content and links to illustrative examples and identified the data source or sources originating the theme.

Following the submission of all preliminary themes for each data segment, unit lead(s) reviewed all entries in the spreadsheet and applied codes to categorize the entries by themes that spanned multiple data segments. The unit lead(s) then sorted the codes to guide discussion during the first of 3 consensus-building meetings that focused on theme generation. The categories with the most entries in the spreadsheet received the greatest dedicated time for discussion, with each analyst sharing their analysis as the theme appeared (or did not) in their assigned data segment or segments. The unit then spent the rest of the time discussing unique or smaller emerging themes.

On the basis of this initial consensus-building meeting, unit lead(s) assembled an outline before the second consensus-building meeting that focused on collaboratively confirming and sorting themes into 3 categories: main, emerging, or continuing and evolving. Main themes were defined as the most pervasive themes identified during that period that appeared to affect vaccine confidence broadly or among a specific group of people. Emerging themes were identified as new, lower-volume themes that were gaining traction or higher-volume themes that did not appear in previous reports. Continuing and evolving themes included topics that had been covered in previous reports but had shifted in nuance or emerged in different data segments from those in which they were originally identified. Lead(s) explained how and why they categorized themes based on the findings and discussion from the theme generation meeting. They also explained why the themes were categorized as main, emerging, or continuing and evolving. Analysts then voiced agreement or disagreement with the categorization by providing additional context and feedback. The lead(s) used the discussion to reorganize and reframe the themes to more accurately represent how they emerged in the data—in some cases dividing or combining preliminary themes. The team finished these meetings by coming to a consensus regarding the final outline of the report. Following the meeting, analysts pulled relevant data for each theme that they had already identified from their data segment or segments and conducted a secondary deep-dive analysis of each of the outlined themes. The analysts collected raw data from all segments into a single document, including illustrative visual and text or quote examples of the themes in social media posts, poll results, and news headlines. Unit lead(s) reviewed all relevant data provided by the analysts on each designated theme for the report and performed additional analysis to uncover nuances within each identified theme.

### Report Production

#### Drafting Theme Narratives

Unit lead(s) drafted a narrative for each theme that described consumer questions, concerns, frustrations, information voids, message penetration issues, and misunderstanding of science or guidance based on their analysis of the pulled data. Narratives also highlighted circulating mis- and disinformation and clarified which demographics appeared to be more affected by that theme if it could be discerned. Each theme’s narrative included links to illustrative examples, the latest literature, and recent polls to provide real-world context to each theme and was crafted through the lens of our analytical frameworks to connect the theme to known behavioral impacts on vaccine confidence, demand, and uptake. Themes determined to be “main themes” had the most substantial narratives, whereas continuing and evolving narratives were limited to a few sentences, focusing on the changes from when that theme was previously featured.

Each theme’s narrative concluded with “ways to take action,” providing concrete recommendations for how communicators, community leaders, health care providers, and health authorities could improve COVID-19 vaccine confidence, demand, and uptake based on evidence-informed practices in behavioral science and communication theory. This section typically included communication, programmatic, and engagement opportunities. The communication actions provided suggestions for filling information voids, correcting message penetration issues through reframing, and engaging specific trusted messengers to disseminate and amplify messages. The programmatic suggestions identified how new or existing programs, processes, or systems could change or be engaged. Finally, research suggestions clarified what research gaps and unanswered questions could be further explored.

When the Insights Unit had 2 leads, each lead was responsible for interpreting and drafting distinct theme narratives. After they completed the process described previously for their assigned themes, they met to discuss and review their findings and proposed actions. When the Insights Unit had 1 lead, the unit lead drafted all the theme narratives and met with the analyst with the most experience to participate in a similar feedback loop before meeting with the larger unit. After a report was fully drafted, a third consensus-building meeting focused on reviewing the narratives with the entire team to ensure content integrity.

#### Applying the Threat Matrix

Following confirmation of each theme’s narrative with the unit, the lead(s) facilitated a discussion to determine the threat level and directionality of each main and emerging theme. Using the threat matrix, the team discussed the level of risk to vaccine confidence (ie, whether the theme would directly or indirectly lead to vaccine refusals and decreased uptake, which groups of people would have their confidence and uptake most affected by the theme, and the volume of spread that the theme appeared to have across data segments and within different groups of people). The team came to a consensus on the level of potential risk to vaccine confidence and uptake for each theme represented: high, moderate, low, or positive. After the risk level was confirmed, we discussed the directionality of the main themes to determine whether the themes were *increasing*, *stable*, or *decreasing* in volume*.*

All emerging themes were classified as increasing due to the nature of the category. The analysts identified the directionality of a theme based on the data from their individual data segments when compared to previous reporting periods. If there was no consensus between the data segments on the directionality of a theme, then we determined the directionality for each data segment and the overall trend across all data segments. For example, if a theme decreased slightly in frequency in the news but increased significantly on social media and CDC-INFO, then we labeled the theme as increasing. Themes that remained at the same volume and spread but had been previously overshadowed by high-risk or high-volume themes and developments across multiple reporting periods relative to each data segment’s baseline were classified as stable in directionality.

#### Assembling the Report

Once all themes were classified through consensus, the order of the report outline was confirmed, with the main theme posing the highest threat to vaccine confidence, demand, and uptake listed first. Subsequently, a written executive summary provided an overview of the main themes identified in the report and the primary ways to act.

### Ethical Considerations

The methodology was determined to be non–human subject research through the CDC’s ethics and project approval process. Additional ethics approval was not required as the data being considered were deidentified during the analysis step. Primary data sets, such as social listening data collected via Meltwater, comprised publicly available data falling under the public domain. Secondary data sets, such as survey data, did not contain any personal identifiable information, and data collection and analysis for those data sets underwent their own independent ethics approval processes.

### Quality Improvement

#### Process Review

The urgency of COVID-19 pandemic and accompanying vaccine confidence issues necessitated creating reports as the methodology was being built. Our methodology underwent continuous process improvement as workforce capacity and contextual changes ebbed and flowed and analysts and unit leads innovated with every report edition. The methodology also underwent several distinct evaluation efforts to increase the rigor of the research process, the validity of the findings, and the usability of the reports.

For example, in June 2021, to identify and reduce threats to the validity of the report’s findings, the Insights Unit team undertook a formal process evaluation outlining the methods used for identifying and classifying themes and assessed the rigor of data collection and analysis. The team evaluated how data were collected, how the themes were categorized, how the prevalence of the data was determined, and how the unit team members determined the threat to vaccine confidence. An evaluation expert interviewed past and current team members, conducted concept mapping exercises with unit leads, observed a full cycle of a report, and reviewed standard operating procedure documents and literature. Through this evaluation, the team was able to identify and articulate the process they had developed to date using established team-based applied qualitative data collection and analysis techniques and best practices. The evaluation also resulted in several changes to the data collection, analysis, and interpretation of future reports, including (1) developing a codebook (Table S1 in Multimedia Appendix 2) and onboarding training to ensure that team members shared a common framework for understanding the data they encountered, (2) expansion of preliminary theme submissions for each data source to include relevant quantitative metrics related to the prevalence of the theme within that data source, (3) reducing reliance on third-party reports by using these reports to triangulate findings from raw data inputs rather than analyzing third-party reports as raw data inputs, and (4) requiring team members to update their data segment’s standard operating procedure at the end of deployment to ensure that their process innovations were passed on to their replacements.

Some evolutions of our methodology were not a result of this evaluation but rather contextual shifts. Data sources included and analyzed for each report varied over time largely because of the increased capacity of the Insights Unit due to more analysts joining the unit over time. This allowed for more personnel time to be dedicated to the analysis of primary data sources, which in turn led the team to identify additional primary sources to use, such as Meltwater and Semrush (Semrush Holdings, Inc). In addition, some of the secondary data sources, mostly insights reports produced by external agencies and organizations, halted their reports over time. This development and the unit’s ongoing use of continuous quality improvement principles decreased the necessity of relying on third-party reports. The evaluation process also led to the creation of a more substantial onboarding process to ensure consistent application of our qualitative and quantitative analysis methods and deductive and inductive approach as outlined in relevant standard operating procedures.

#### Expert Review

In July 2021, an external subject matter expert with a background in infodemics, fact checking, and digital media was contracted to conduct a Strengths, Weaknesses, Opportunities, and Threats analysis of the Insights Unit and its reports. The expert conducted in-depth interviews with leads and analysts and observed the process of producing a report from inception to publication. The analysis found that the methodology used and subsequent reports were unique in terms of diverse subject matter expertise of the unit membership, speed and regularity, constant reflection, and scale (ie, considering the whole information ecosystem, including listening for unanswered questions and topics leading to confusion, as well as mis- and disinformation and the use both of many different data sources and data sources unique to the CDC, such as CDC-INFO). However, the analysis also found that the reports lacked a clear objective as they attempted to serve as an early warning system for misinformation, a rapid response system to help manage official communications, and a means to identify content gaps. The reports also had a wide target audience and lacked mechanisms to ensure intercoder reliability as it relied on a “lone wolf” (ie, single coder) coding strategy through unit lead(s). The review found additional weaknesses to address, such as the need to clarify definitions and the codebook so that it was uniformly applied and the challenges of comparing data from disparate sources with widely varying denominators. Steps were taken in the second year of the Insights Unit to ensure that the codebook was uniformly applied through enhanced onboarding training for Insights Unit deployers and staff while expanding the “lone wolf” coding strategy by increasing the number of staff members trained.

## Results

### Production and Distribution

As of August 2022, the Insights Unit has produced 27 full reports, 5 rapid reports, and 2 special reports. Rapid and special reports used the same methodology applied to full reports except with a shorter data collection period. Rapid reports pertain to a specific and time-sensitive issue, such as the emergency use authorization of COVID-19 vaccines in adolescents.

SoVC reports were distributed via email to a listserve of 956 readers, including CDC staff and external partners (eg, federal agencies, professional associations, community-based organizations, and nonprofits, in addition to worldwide health authorities and government representatives). Reports were also published on the web and have been downloaded 22,361 times. Findings and action steps were also shared through presentations to local, national, and international stakeholders from March 2021 to December 2021.

### Use

Within the agency, the reports were used by VTF and the emergency operation center to directly inform strategies for communication and community engagement. VTF’s communication team used the reports to enhance the CDC’s COVID-19 vaccine social media content, update digital content on myths and frequently asked questions, and improve search engine optimization. As the reports highlighted challenges faced by specific demographics, VTF staff used the findings to support partner organizations and state and jurisdiction efforts for on-the-ground vaccine demand generation. The reports also served as a unique mechanism for providing feedback to VTF’s vaccine distribution teams by highlighting underlying practical issues impacting vaccine uptake, such as challenges navigating digital systems to schedule vaccine appointments.

Between March 2021 and August 2022, SoVC reports were referenced by groups outside the CDC 22 times on the web. Organizations and agencies who referenced the reports included state and local health departments (n=6), nonprofit organizations (n=5), professional associations (n=3), and congressional committees (n=2). The reports were mostly referenced as a resource for learning about COVID-19 vaccine confidence and demand, with nearly half of the identified references (n=11) linking directly to the reports. Many of these references focused on increasing uptake of the COVID-19 vaccine, sharing the latest information on COVID-19 vaccinations, and improving public health communications. SoVC reports were also cited by research studies (n=4) to justify study goals and findings. For example, one journal article used a report as context to describe how perceptions of vaccine effectiveness can impact vaccine confidence [16-19].

In addition to serving as an informative resource, the reports were used to demonstrate how the CDC monitored and responded to COVID-19 vaccine mis- and disinformation. Then CDC director Rochelle Walensky referenced the reports and the Insights Unit at 2 congressional hearings, describing how the CDC was working to understand the public’s perceptions of COVID-19 vaccines and address the public’s information needs to increase vaccine uptake [20]. Furthermore, we identified 2 sources that applied findings and themes from the reports, one of which was the Africa Infodemic Response Alliance, which issued a report in June 2021 on COVID-19 infodemic trends containing recommendations adapted from an SoVC report.

### Evaluation

To better understand the consumers of the reports, the value consumers derive from them, and how to further improve the reports’ utility, we conducted an outcome evaluation in October 2021 via a survey of the audience. We received 53 survey responses (6.4% response rate from the email distribution list) and solicited feedback from 6 stakeholders and 1 focus group session with 4 Insights Unit staff members. We found that approximately 75% (40/53) of respondents said that they found the reports “very” or “extremely” relevant and 52% (32/61) of respondents used the reports to inform communication strategies. The respondents also made suggestions for improvements (eg, creating bulleted summaries of findings and providing resources for readers to operationalize the reports’ recommendations), which led to several changes to make the reports more readable and useful. Subsequent reports, beginning in early 2022, incorporated bulleted summaries of themes rather than descriptive narratives and resources. These were provided within the reports themselves, and links to additional resources for communicating about the themes were also provided.

## Discussion

### Informing Action

Our approach and methodology intentionally responded to urgent, interconnected needs. The primary priority was to assist the agency and our partners in acting more swiftly to address the questions, concerns, perceptions, information voids, and circulating mis- and disinformation regarding COVID-19 vaccines and inform communication and programmatic action. The second priority was to establish a coordinated mechanism for collecting, reviewing, and synthesizing qualitative and quantitative data from multiple internal and external sources. Our unit and reports were intended to bridge the gap among the CDC’s current social listening efforts, reports produced by external organizations, and current surveys and polls conducted by the CDC and third parties. Being one of the first integrated infodemic management products of such scale, scope, and regularity to be produced at the CDC, these reports interpreted findings in a novel way through the lens of behavioral theory and public health best practices. Similar approaches often focus on singular data streams rather than multiple data inputs to contextualize findings.

While our methodology was developed specifically in the context of understanding vaccine confidence and demand and the information ecosystem regarding COVID-19 vaccines in the United States, it can be adapted for any public health issue. Furthermore, the varied use of our reports suggests that they met the information needs of a diverse audience, served an informative purpose, and conveyed an urgency to address information challenges, implying that similar reports on other public health topics would support both routine and emergency functions. However, this methodology should not be directly reproduced without contextualization. It is important to conduct needs assessments and landscape analyses to understand the current state of the topic at hand and the population that is going to be examined, in addition to identifying data sources for consideration. Irrespective of these contextualization needs, our integrated analysis and the consensus-building processes are replicable, and others could create similar reports. In addition, the reports were produced by a rotating staff with different backgrounds and changing capacities, which implies that the analysis process and techniques used can be learned and scaled up or down depending on the skills and bandwidth of the staff.

### Limitations

There were several limitations to this methodology both for generating insights themselves and regarding its utility.

#### Data and Granularity

A limitation of the SoVC reports themselves was the data sources used and their inherent limitations. As widely documented, data gleaned from social media may present a skewed picture of the population of interest due to a lack of representativeness. For instance, people of certain age groups, languages, or geographic locations may be over- or underrepresented in social media data depending on how digitally connected they are [9,21,22]. Demographic biases in the data are also likely further pronounced by the limited availability of data from closed social media (eg, private groups on WhatsApp, Telegram, or Facebook) or the dominance of more easily accessible data (eg, X [formerly known as Twitter] conversations) in our analyses [3,23]. Furthermore, demographic data are not always linked to user-generated content on social media [21]. Sophisticated artificial intelligence and algorithms would be needed to deduce user demographics based on profile content generation and digital footprints, which could present ethical challenges. In addition, users may present themselves differently in online spaces than in face-to-face interactions; therefore, their content may not always be an accurate proxy for their true intentions, perceptions, or behaviors [9]. This makes it difficult to detect local contexts or nuances in large data sets compiled for national-level analysis [3]. For example, data aggregator tools such as Google Trends exclude smaller trends (ie, searches made by very few people) [24]. While we have tried to minimize the impact of some of these limitations by integrating multiple data sources beyond aggregated social media or web analytics data, they remain important to bear in mind when interpreting findings. One potential way to mitigate this issue is to conduct a comprehensive analysis of all data sources before inclusion to better understand the demographics being sampled and ensure that the data sources accurately reflect the demographics of the population of focus. Another potential tactic would be to establish thresholds for specific key terms for each data source to help identify when mentions are above the baseline for each topic. Research should also be conducted to explore what type of infodemic management is being done at subnational and local levels to identify emerging best practices to test and validate.

#### Potential Bias and Subjectivity

Social listening and analysis tools, especially focused on the digital space, have primarily been built for the purposes of marketing and external communication campaign tracking. Adapting such tools for use in the public health space can be challenging, and in the case of our methodology, we had to adapt tools to meet our analytical needs. In addition, there are no systematic, near–real-time approaches to consider the risk of a qualitatively identified theme in the context of a behavioral health model. This was further complicated by the lack of indicators to directly link exposure to information ecosystems and vaccine refusal. We attempted to mitigate the impacts of subjectivity through consistent training, which involved overlap between deployers to ensure adequate knowledge transfer and making decisions based on consensus. While consensus-based decision-making is not perfect, it did reduce the potential impacts of individual bias on assessing the threat of themes to vaccine confidence. Those considering adapting these methods for their own use should consider how they could further reduce potential bias and ensure that theoretical frameworks are deployed. Furthermore, artificial intelligence and machine learning could potentially be leveraged to compliment the human component of our process to reduce the bias and subjectivity of analysis by performing the initial analysis of raw data sources.

#### Adaptability

The methodology itself may also be limited in its ability to be operationalized in different public health settings. First, the scale of the unit and process described in this manuscript required several dedicated staff members and significant personnel time. This may not be accessible or replicable outside of an emergency response. Research needs to be conducted to better determine ways to adapt this process for routine use. Furthermore, the process may not be achievable exactly as described for most health authorities until tools are developed and deployed to reduce the amount of personnel time required to generate insights. Different iterations of this methodology should be designed, tested, and evaluated in different public health settings to determine minimal and optimal staffing, funding, and mechanisms. Researchers should consider how to leverage artificial intelligence and related technology in the collection and analysis of these types of activities and reports.

#### Impact and Outcomes

Additional evaluation is also needed to better understand the utility of rapid or deep-dive reports compared to standard reports and how the different types of reports could be used more impactfully. In addition, because of how the unit was established, we did not have decision-making authority in acting based on the reports’ findings and recommendations. Units such as this one should be integrated into programs, and mechanisms should be put into place to maximize the use of the reports.

The impact of these reports on program and campaign activities leading to improvement in vaccine coverage also needs to be assessed. Limited indicators exist to directly link exposure to information ecosystems or communication campaigns and vaccine uptake and coverage. The guiding analytical frameworks we used do not currently include information ecosystems and campaigns in their models. Furthermore, most sociobehavioral measures related to vaccination typically measure intent to vaccinate but cannot directly link to whether behavior was completed. Identifying indicators that more directly link action with exposure to infodemics, communication campaigns, and interventions is necessary to better understand their impact on behaviors as well as establish best practices. Indicators outside of public health should be considered for adaptation, tested, and validated in the field.

### Conclusions

The Insights Unit’s methodology is not a solution for infodemics but rather a diagnostic technique that can rapidly identify opportunities for intervention. In addition, identifying mechanisms to act on the findings need to be created at the onset to ensure success. This methodology is also an adaptable process that can be scaled and used in a variety of public health settings for all public health issues. It can be applied beyond acute public health crises, such as within routine monitoring and surveillance. The methodology as outlined provides a road map for organizations focused on improving public health to conduct an integrated analysis of disparate data sources to generate and leverage insights during outbreaks and routine programming to inform population-level health. The insights generated can inform the deployment of effective interventions to fill information voids and counter misinformation, including partnering with trusted messengers.

The results of integrated analyses can be considered within already established, evidence-based conceptual frameworks and leveraged to deliver timely and responsive communication, programming, and policies. The continual listening and feedback process of insights units such as the one detailed in this paper allows health authorities to clarify guidance and improve programmatic quality in near real time based on changing public needs, especially within the context of a rapidly evolving public health emergency. Institutionalizing such units could enable public health messages, guidance, programs, and policies to better resonate and meet the information needs of the public. More responsive public health action could build trust between public health authorities and the public, which could lead to higher sustained uptake of health promotion strategies.

Public health authorities are especially well positioned to use their staff, including behavioral and social scientists, communication scientists, informatics specialists, ethicists, and epidemiologists, to institute insights units for population health promotion. These units can be on the frontlines of not only identifying and diagnosing infodemics but also building and promoting the science to test, evaluate, and scale promising interventions to rapidly respond to community needs, counter misinformation, bolster resiliency, and reinforce trust [25]. These units could benefit from being closely connected to traditional public health functions both within an institution and with external partners, including those focused on public health surveillance, field epidemiology, and health communications, to further contextualize and validate data trends [26,27]. Such collaborations allow health authorities to work more proactively.

The methodology and work of the Insights Unit and similar units are critical to the burgeoning field of infodemic management. This methodology has centered on data within the United States, a high-income and predominantly English-speaking country; these methods should be extended to better incorporate non–English-language digital listening in the future because information ecosystems are not limited by language or geographic borders. The methodology presented in this paper provided a national view of vaccine confidence and demand issues; localized subnational applications will require improved digital listening tools and investigation to determine the best online and offline sources from which to collect data. In addition, the development of new technology and platforms at low cost is essential to moving this work beyond high-income countries that can adapt current tools to perform these social listening and analysis functions. Artificial intelligence and machine learning could help reduce the burden of data collection and preliminary analysis, with promising approaches already being tested, such as using machine learning to help identify bots and measure infodemic risk [28]. Finally, more research needs to be conducted to better understand the impact that infodemics and infodemic management have on health behaviors. Indicators should be explored and examined to allow for quantifiable measurement of the burden of infodemics, which in turn will allow for improved evaluation of interventions and infodemic management itself.

## Appendix

Multimedia Appendix 1 Example from State of Vaccine Confidence report 11—process from data segment analysis to final outline. This figure highlights the steps conducted for integrated analysis of each report. This figure contains actual data from the 11th report. Step 1 represents the preliminary themes identified by data segment. Step 2 represents the themes submitted in step 1 sorted by the codebook to look for congruence. Step 2’s results help guide step 3’s consensus-building meeting, which is used to draft a report’s outline in step 4. During step 5, the Insights Unit reviews the outline developed in step 4 and, once confirmed, returns to their data sources to pull relevant samples of data based on the themes in the outline. In step 7, reports are drafted using the data collected in step 6, and the report is reviewed with the team to ensure accuracy and assess threat level. In step 8, a report outline is refined, and the report is finalized and submitted to clearance.

Multimedia Appendix 2 COVID-19 State of Vaccine Confidence codebook. A qualitative codebook was established because of our process evaluation that broke up our themes into various codes and developments. The description column in Table S1 provides the inclusion criteria for each development.

Multimedia Appendix 3 COVID-19 State of Vaccine Confidence Insights Unit Boolean strings. An example of the Boolean string used in Meltwater to collect news and social media mentions. All strings used the “Base Boolean String,” and “substrings” were added using the “AND” operator to help dive deeper into the subtopics of interest.

## Abbreviations

CDC: Centers for Disease Control and Prevention
SoVC: State of Vaccine Confidence
VTF: Vaccine Task Force
