# Supplementary figures and images for: Infodemics and Vaccine Confidence: Protocol for Social Listening and Insight Generation to Inform Action
Source: JMIR Public Health Surveill. 2024 Oct 24;10:e51909. doi: 10.2196/51909 (PMC11544329; doi:10.2196/51909)

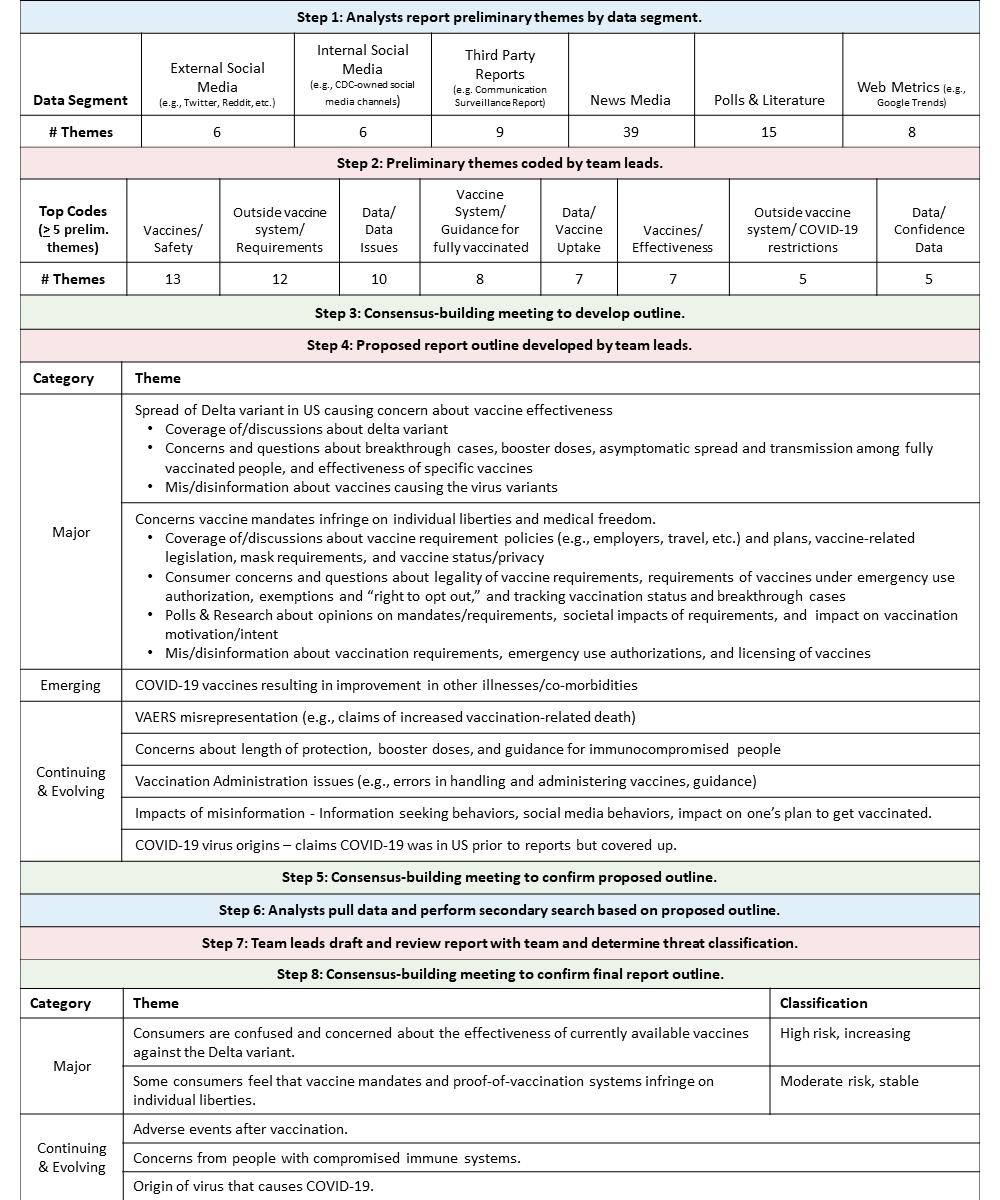

Supplement: Multimedia Appendix 1 [file publichealth_v10i1e51909_app1.png]
