# Supplementary material for: Infodemics and Vaccine Confidence: Protocol for Social Listening and Insight Generation to Inform Action
Source: JMIR Public Health Surveill. 2024 Oct 24;10:e51909. doi: 10.2196/51909 (PMC11544329; doi:10.2196/51909)
Supplement: Multimedia Appendix 2 [file publichealth_v10i1e51909_app2.docx]

**Table S1.** COVID-19 State of Vaccine Confidence Code Book^^[[1]](#footnote-1)^^

A qualitative code book was established because of our process evaluation that broke up our themes into various codes and developments. The description column describes inclusion criteria for each development.

| **Code** | **Development** | **Description** |
| --- | --- | --- |
| Data | Data (general) | 1. Any data related to vaccination or vaccination research |
|  | Data Issues | ● Lack of data  ● Data inequities  ● Unsettled science or contradictory data  ● Lack of data disaggregation (e.g., by ethnic group)  ● Lack of specific data (e.g., specific vaccination outcomes.)  ● Misinformation about:  ○ VAERS data  ○ Debunked data or studies |
|  | Supply and demand data | 1. Allocation/availability of vaccines by state/county 2. Demand outstripping supply 3. Supply outstripping demand 4. Excludes vaccine shortages or wastage |
|  | Coverage/uptake data | 1. Vaccine uptake/vaccine coverage data 2. Vaccine series completion data |
|  | Confidence data | 1. Polls and other data on elements of vaccine confidence (e.g., intention to vaccinate or how events affected confidence) |
|  | Non-vaccine data decreasing urgency to vaccinate | 1. Non-vaccination research/data that may decrease urgency to vaccinate (e.g., declining COVID-19 cases and deaths or studies showing lower severity illness on certain groups) |
|  | Non-vax data increasing urgency to vaccinate | 1. Data unrelated to vaccine safety and effectiveness (e.g., COVID-19 risk data and negative psychological and socioeconomic effects of the pandemic) |
| Vaccines | Vaccines (general) | 1. Verified developments relating to the vaccines themselves (e.g., vaccine effectiveness and safety) 2. Any developments that damage the safety/effectiveness reputation of vaccines have an outsized impact on intentions to vaccinate. |
|  | Effectiveness | 1. Vaccine effectiveness data in general, for specific populations (e.g., immunocompromised individuals), or against new variants (e.g., Alpha, Delta, etc.) 2. Discussions that affect the perception of effectiveness (e.g., breakthrough infections, need for booster doses, etc.) 3. Discussions of administration affecting effectiveness (e.g., mixing of vaccine brands, longer than recommended lag between doses, etc.) 4. Discussions of vaccine effectiveness compared to "natural immunity" from a previous COVID-19 infection 5. Misinformation about effectiveness of vaccines. |
|  | Safety | 1. Positive and negative developments/data about safety of COVID-19 vaccines 2. Perceptions of safety, including during and post-vaccination discomfort, side effects, and adverse events 3. Misinformation about adverse events after vaccination; safety concerns for specific groups of people or for vaccine ingredients |
| Vaccinators | Vaccinators (general) | 1. Institutions and people involved in administering vaccines (e.g., doctors, nurses, etc.) 2. Vaccination sites (e.g., clinics, mass vaccination sites, etc.) 3. Organizations and entities (e.g., pharmacy retail programs, hospitals, etc.) focused on vaccination administration 4. Non-traditional vax sites (e.g., schools, religious spaces, etc.) |
|  | Trusted vaccinators | 1. Recommendations to get vaccinated from trusted healthcare providers (e.g., doctors, nurses, and healthcare workers) 2. Stories of trusted vaccinators recommending and administering vaccines 3. Expressed hesitancy among trusted vaccinators |
|  | Practical issues | 1. Issues accessing vaccination (e.g., challenges in booking appointments or physically getting to appointments) |
|  | Inequity in vaccine access | 1. Stories specifically about differences in vaccine access for specific groups |
|  | Administrative errors | 1. Concerns about stories of errors made by the vaccinators (e.g., administering incorrect doses, incorrect duration between doses, improperly storing or refrigerating vaccines, etc.) 2. Does not include intentional errors (e.g., June 2021 story of a pharmacist tampering with 500 vaccines) |
|  | Unfilled appointments | 1. Issues of underutilization of available vaccines |
| Vaccine System | Vaccine system (general) | 1. Institutions, policies, and processes that bring vaccines into being and distribute them to the population (excluding vaccines and vaccinators) |
|  | Research and development | 1. Creation of vaccines (e.g., vaccine development, clinical trials, booster dose development, etc.) 2. Does not include stories about the need for additional testing for different populations that were inadequately represented in clinical trials |
|  | Licensing and authorization | 1. Developments around the licensing, authorization, and continuous safety tracking/monitoring of the vaccines (e.g., emergency use authorization, advisory panels for CDC and FDA, etc.) 2. Does not include misinformation about VAERS |
|  | Supply | 1. Manufacturing to distribution 2. Storage and handling 3. Vaccine shortages 4. Vaccine wastage and disposal |
|  | Demand generation and barriers | 1. Developments that aim to increase demand for vaccines (e.g., incentives) 2. Does not include expansion of vaccination efforts |
|  | Official guidance | 1. Discussion or reports of official guidance from trusted public health agencies (e.g., CDC, WHO, etc.) 2. Guidance for vaccination and people who are fully vaccinated |
|  | Malpractices | 1. Stories of malpractices within the vaccine system can damage its reputation and reduce the trust that the public places in its institutions to do the best by public health and not by some other interest like profit and to act from a place of care rather than from prejudice and bias. Includes both new and past mistakes and bad practices. |
|  | Conspiracies | 1. Claims about government conspiracies, “Big Pharma conspiracies”, and previously debunked malpractices |
| Outside Vaccine System | Outside vaccine system (general) | 1. Actions taken by economic entities, such as businesses or employers, influencers, and the general public 2. Developments within digital media where powerful rhetoric can become a development all on its own |
|  | Requirements | 1. Policies about vaccination requirements (e.g., employee or school requirements) 2. Proof of vaccination systems (i.e., "vaccine passports") 3. Travel requirements |
|  | Economic closing or re-opening | 1. Restrictions to slow the spread of COVID-19 2. Lifting of COVID-19 restrictions and “return to normal” |
|  | Public behaviors | 1. Actions that support or undermine vaccinations in others (e.g., fake vaccine scams, vaccine-card fraud, etc.) 2. Discussions of vaccine (brand) "shopping", "vaccine hunting", and "vaccine tourism” 3. Violence over masking or vaccination requirements 4. Social norms among the vaccinated (e.g., continuing to wear masks and social distancing post-vaccination) or among unvaccinated |
|  | Misinformation policing | 1. Stories and discussions of digital company policies to remove or flag problematic content or users |
|  | Influencer narratives | 1. Discussions by prominent public figures that may support or undermine vaccine confidence (e.g., expert critiques, the lack of intention to get vaccinated by prominent politicians or celebrities, etc.) 2. Stories or discussions of getting vaccinated |
|  | Public narratives | 1. Discussions by members of the public that either support or undermine vaccine confidence (e.g., vaccine selfies, personal accounts of magnetism or adverse effects, public vaccine refusals, etc.) 2. Persistent anti-vaccination tropes (e.g., medical segregation, experimentation, etc.) |
|  | Cultural clashes | 1. Discussions of mandates impinging on individual freedoms and lack of vaccination as a sign of resistance to being controlled 2. May include broader tropes brought in to justify vaccinations or decisions not to vaccinate as being rooted in cultural ideas about the role of government, business, or science in society |
|  | COVID-19 denialism | 1. Tropes that underplay the seriousness of COVID-19 (e.g., denying that it exists, arguing that only certain people are vulnerable, spread is exaggerated, etc.) 2. Discrediting preventive measures to control COVID-19 (e.g., masking, social distancing not effective) 3. Does not include discussions about vaccines providing protection |
|  | Unverified COVID treatments | 1. Information claiming that COVID-19 can be prevented or treated by unverified treatments or that because of such treatments, vaccines are not necessary 2. Includes unverified or debunked claims that certain herbs, essential oils, or chemicals can treat COVID or reduce its symptoms or their severity. |

1. Inclusion criteria for all codes required development or finding to have occurred during the reporting period specific for a single report. [↑](#footnote-ref-1)
