# Supplementary material for: Infodemics and Vaccine Confidence: Protocol for Social Listening and Insight Generation to Inform Action
Source: JMIR Public Health Surveill. 2024 Oct 24;10:e51909. doi: 10.2196/51909 (PMC11544329; doi:10.2196/51909)
Supplement: Multimedia Appendix 3 [file publichealth_v10i1e51909_app3.docx]

#### **Table S2:** COVID-19 State of Vaccine Confidence Insights Unit Boolean Strings

Caption: Below is an example of the Boolean string used in Meltwater to collect news and social media mentions. All strings used the “Base Boolean String” and “sub-strings” were added using the “AND” operator to help dive deeper into the subtopics of interest.

| **Base Boolean String^[[1]](#footnote-1)^** | |
| --- | --- |
| (((("vaccine" OR "vaccination" OR "vaccines" OR “vaccinate” OR "immunization" OR "immunizations" OR "immunize" OR "vax" OR "vaxx" OR "shot" OR "shots" OR "va((ine" OR "va((!ne" OR "#vaccine" OR "vaccinated" OR "vacine" OR  "vaccin" OR "vacines") AND ("COVID-19" OR "COVID19" OR "COVID" OR "Coronavirus" OR "Corona" OR "China virus" OR “Chinavirus” OR “Chinese virus” OR “Wuhan virus”)) OR ("covid19vaccine" OR "covidvaccine" OR "covidvax" OR "coronavaccine" OR "covid19vaccines")) | |
| **Selected sub-strings^[[2]](#footnote-2),^^[[3]](#footnote-3)^** | |
| Effectiveness | ("effective" OR "effectiveness" OR "effectivness" OR "efficacy" OR "transmission" OR “transmit” OR "spread" OR "prevent" OR "prevents")) |
| Side Effects | ("side effect" OR "side effects" OR "pain" OR "fever" OR "headache" OR "redness" OR "red" OR "sore" OR "fatigue" OR "exhausted" OR "sleepy" OR "muscle" OR  "aches" OR "chills" OR "tiredness" OR “symptom” OR “tired” OR “headache” OR “nausea” OR “nauseous”)) |
| Adverse Events | ("damaged" OR "injured" OR "maimed" OR "bells palsy" OR "bell's palsy" OR "bells' palsy" OR “bellspalsy” OR "injury" OR "blood disorder" OR "covid arm" OR "covidarm" OR "paralyzed" OR "paralyze" OR "adverse" OR "ruin" OR "seizure" OR "seizures" OR “faint” OR “hospital” OR “allergic reaction” OR “paralysis” OR “paralyzed” OR “unresponsive” OR “severe reaction” OR “VAERS”)) |
| Fertility/Pregnancy | ("pregnancy" OR "pregnant" OR "miscarriage" OR "fertile" OR "fertility" OR "fetus" OR "fetal" OR "birth" OR "defect" OR “unborn” OR “womb”)) |
| Access | ("appointment" OR "appointments" OR "appt" OR "appts" OR "schedule" OR "sign up" OR "register" OR "slot" OR "time" OR "scheduled" OR "scheduling" OR "registering" OR “cancel” or “access”)) |
| Natural Immunity/Previous Infection | (“previously infected” OR “natural immunity” OR “recovered”)) |
| Safety | (“safe” OR “safety” OR “protect” OR “protected” OR “prevent”)) |
| Variants | (“variant” or “strain” or “variants” or “varients” or “ varient” or “B.1.617” or “P.1” or “B.1.351” or “B.1.1.7” or “UK variant” or “india variant” or “Indian variant” or “brazil variant” or “south Africa variant” or “SA variant”)) |

1. Used for all search strings. [↑](#footnote-ref-1)
2. Added to base string via AND operator. [↑](#footnote-ref-2)
3. Note: Search strings evolved over time. [↑](#footnote-ref-3)
